# Olfactory Tuft Cells Are Critical to Basal Inflammation, Innate Immune Response to Viral Infection, and Modulation of Quiescent Stem Cell Activation, Proliferation and Differentiation

**DOI:** 10.1111/cpr.70281

**Published:** 2026-09-19

**Authors:** Sai‐Sai Zhang, Haiyan Wang, Yi‐Sen Yang, Yi‐Hong Li, Defu Yu, Yushi Yao, Ruhong Zhou, Liquan Huang

**Affiliations:** ^1^ College of Life Sciences, Zhejiang University Hangzhou Zhejiang China; ^2^ Institute of Immunology and Sir Run Run Shaw Hospital, Zhejiang University School of Medicine Hangzhou Zhejiang China

**Keywords:** Gγ13, H1N1 influenza virus, olfactory tuft cells, quiescent stem cell, signal transduction, Trpm5

## Abstract

The olfactory mucosa serves as both a sensory organ and an immune barrier to protect against bacterial and viral invasion and other insults. It is unclear how different types of olfactory mucosal cells coordinate and contribute to these two functions. We set out to reveal the critical roles of a subset of microvillous cells of the mucosa, olfactory tuft cells, in protecting and reconstructing this vital olfactory sensory organ. We first validated the expression of canonical gustatory signalling proteins and other molecular markers in olfactory tuft cells. Genetic disruption of the *Gng13* and *Trpm5* genes that encode the two gustatory signalling proteins, G protein subunit Gγ13 and transient receptor potential ion channel Trpm5, respectively, resulted in elevated basal inflammation and enhanced activation of the quiescent stem cells—horizontal basal cells (HBCs) in the mucosa. Nasal infection of H1N1 influenza virus further exacerbated the inflammation and delayed the resolution of inflammation in the mutant mucosa, including more immune cell infiltration, augmented cytokine production and cell death, increased HBC proliferation and direct differentiation into tuft cells, and prolonged olfactory tuft cell hyperplasia. Cytokine treatment of the cultured olfactory epithelial organoids indicated that the cytokines that were found to be elevated in the mutant mucosa, including interleukin‐4 (IL‐4), IL‐13 and interferon‐γ (INF‐γ), are able to stimulate HBC activation. Together, our results indicate that olfactory tuft cells play an important role in maintaining the baseline inflammation under the steady‐state condition, and altering inflammatory magnitude and modulating HBC activation and differentiation in the olfactory mucosa following the viral infection. Our findings shed light on new roles of olfactory tuft cells in innate immune response, quiescent stem cell activation and neuroimmune interactions, and provide novel therapeutic targets for preventing and treating stem cell‐related olfactory disorders such as chronic rhinosinusitis and long COVID.

## Graphical abstract

Olfactory tuft cells communicate with other mucosal cells in maintaining olfactory epithelial immunity and sensory function. Disruption of tuft cell signalling pathways causes a skewed immune response, leading to heightened inflammation and increased activation of the quiescent stem cells, which are further exacerbated by viral infection.

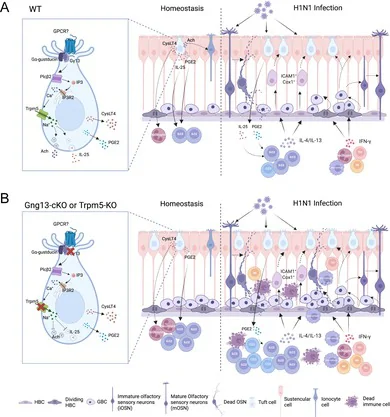

## 1 Introduction

The olfactory mucosa, situated within the nasal cavity and continuously exposed to the external environment, serves two critical physiological roles. Firstly, it functions as the peripheral sensory organ of the olfactory system, enabling the detection of airborne odorants. Secondly, it acts as a frontline mucosal immune barrier, protecting the brain and lower respiratory tract from environmental insults. Compromise of this barrier can result in severe outcomes, including pulmonary infections, neurological dysfunction or even mortality [1]. Despite its dual significance, the mechanisms by which these sensory and immunological functions are integrated, maintained in homeostasis or disrupted following pathogenic invasion and repaired during recovery remain largely unclear [2].

Anatomically, the olfactory mucosa comprises an avascular neuroepithelium, the underlying lamina propria, and the cribriform plate. The neuroepithelium is packed with olfactory sensory neurons (OSNs) and supportive sustentacular cells, interspersed with Bowman's glands and two specialized microvillous cell types: olfactory tuft cells and ionocytes [3, 4]. Underneath these cells reside two stem cell populations: actively proliferating globose basal cells (GBCs), which replenish senescent or damaged epithelial cells, and quiescent horizontal basal cells (HBCs), which are activated only following severe epithelial injury [5]. Each mature OSN expresses a single type of olfactory receptor on its ciliary membrane and transmits odorant signals via its axon to the glomeruli of the olfactory bulb. This highly efficient one‐neuron connection between the nasal epithelium and the brain underpins the remarkable sensitivity of the olfactory system. However, it also establishes a direct anatomical conduit that may be exploited by external pathogens to access the central nervous system (CNS) [1].

The olfactory mucosa is continually exposed to a wide range of environmental agents, including microbiota, viruses, allergens and both organic and inorganic insults. Certain amoebae and bacteria can infiltrate and damage the olfactory neuroepithelium, subsequently travelling along the olfactory nerve to the brain, thereby causing severe pathological outcomes [6, 7, 8]. Numerous viruses, such as SARS‐CoV‐1, SARS‐CoV‐2 and influenza, are known to be olfactotropic and can infect various cell types within the olfactory mucosa, including sustentacular cells and OSNs [9, 10]. Moreover, human cytomegalovirus (HCMV) has been shown to exploit the human olfactory receptor OR14I1 as a cellular entry point into OSNs, suggesting that certain olfactotropic pathogens may hijack the molecular and cellular machinery of the olfactory system to gain access to the CNS [11].

On the other hand, the olfactory mucosa has evolved multiple defence strategies to eliminate infectious pathogens and prevent their spread to the CNS or the lower respiratory tract. For instance, Bowman's glands secrete special antimicrobial mucins [12], while apoptosis and non‐lytic clearance pathways help eliminate infected cells or intracellular viruses to limit inflammation [9, 13, 14, 15]. Even in the absence of inflammation, a small population of resident immune cells—including two distinct macrophage subtypes—is present in the olfactory mucosa, where they may phagocytose infected cells and debris or release cytokines that act on neighbouring immune and epithelial cells [16]. Additionally, a blood‐olfactory barrier (BOB) has been proposed. This barrier comprises tight junctions between apical sustentacular cells, olfactory ensheathing cells (OECs) surrounding the OSN axon bundles, and the endothelial cells of the blood and lymphatic vessels in the lamina propria [17, 18]. Functionally, the BOB may serve as an extension of the blood–brain barrier (BBB), limiting the access of circulating agents and pathogens to the CNS via the olfactory route. However, it also restricts the entry of large molecules, including serum antibodies, into the olfactory mucosa, creating a blind spot in mucosal immune defence. Unlike the CNS, the olfactory mucosa is constantly exposed to the external environment, and some pathogens such as amoebae, bacteria and viruses can damage the BOB structure and infiltrate the mucosa [6, 7, 8, 9, 10, 11]. Furthermore, the BOB structure is vulnerable to infection by olfactotropic pathogens even in vaccinated or previously infected individuals [1]. Clinical reports of influenza‐associated encephalopathy/encephalitis [19] highlight the need to enhance the antiviral defence mechanisms within the olfactory mucosa to protect the CNS. In immunodeficient mouse models and patients, viruses such as influenza and SARS‐CoV‐2 can penetrate the CNS via the olfactory route and disseminate to the interconnected brain regions, triggering neuroinflammation and, in some cases, neurobehavioural deficits or psychiatric manifestations [20, 21, 22, 23, 24]. Interestingly, intranasal inoculation appears to stimulate olfactory epithelial cells to produce homing signals that recruit immune cells to the site. However, the molecular mechanisms underlying the generation and regulation of these homing signals remain poorly understood.

Distinct populations of olfactory epithelial and immune cells appear to play specialized roles at different stages of the host response to pathogen invasion—ranging from the initiation of inflammation to its subsequent resolution and tissue repair [9]. For instance, sustentacular cells express interleukin‐33 (IL‐33), thereby promoting type 2‐mediated inflammation and HBC activation [10, 25], while neutrophils contribute to neuronal apoptosis during the early phase of inflammation but subsequently transition to support neuroprotection and tissue repair [26].

Olfactory tuft cells are a rare, specialized epithelial population sparsely distributed at the apical surface of the olfactory neuroepithelium. They share a core set of genes with other tuft cells, including those encoding the transcription factor Pou2f3, G protein subunits Gα‐gustducin and Gγ13, PLCβ2, transient receptor potential cation channel Trpm5 and choline acetyltransferase (ChAT) [27, 28, 29, 30, 31, 32, 33, 34]. Tuft cells have also been found in other tissues and organs—including the gastrointestinal tract [34, 35, 36], pancreas [37], respiratory airway tract and lung [38, 39], gallbladder [40] and thymus [41]—where they contribute to the detection of luminal stimuli such as viruses, bacteria, protists and helminths [34, 35, 42, 43, 44] and promote type 2 immune responses [34, 35, 45, 46, 47]. In the olfactory mucosa, tuft cells have been implicated in the allergen‐induced olfactory stem cell activation [4, 29]. However, their exact role in mucosal inflammation and olfactory epithelial repair remains to be elucidated.

In this study, we disrupted gustatory signalling pathways by selectively ablating the expression of Gγ13 and Trpm5 in olfactory tuft cells, and found that the genetic perturbations led to elevated basal inflammatory states, increased cytokine expression, and following H1N1 influenza infection, the mutant animals showed altered basal inflammation, heightened and prolonged immune responses, and enhanced activation of the quiescent stem cells.

## 2 Materials and Methods

### 2.1 Experimental Design

This study was designed to investigate the possible roles of olfactory tuft cells in maintaining the immune defence of the olfactory mucosa under normal conditions, in regulating innate immune response to H1N1 influenza virus infection, as well as in controlling the activation of the quiescent stem cells—horizontal basal cells (HBCs) and subsequent tissue repair. We explored the inflammation states of both WT and mutant mice with the expression of the canonical gustatory signalling proteins Gγ13 and Trpm5 abolished in olfactory tuft cells before and after H1N1 infection, and examined the changes in immune cell infiltration, cytokine expression, cell death, olfactory tuft cell hyperplasia and HBC proliferation and differentiation in the mucosa. We used the olfactory epithelial organoids to assess the cytokines' action on HBC activation.

### 2.2 Animals

C57BL/6 mice were purchased from the Shanghai SLAC Laboratory Animal Company. ChAT‐IRES‐Cre (hereafter referred to as ChAT‐Cre), Trpm5‐conventional knockout/lacZ knockin (heterozygous Trpm5‐lacZ^+/−^, homozygous Trpm5‐lacZ^+/+^ or Trpm5‐KO), Pou2f3‐CreERT2‐IRES‐EGFP knockin, Ai9 and Ai14 mice (Jax stock numbers 006410, 005848, 037511, 007909 and 007914, respectively) were obtained from the Jackson Laboratory. The Gng13^flox/flox^ mice were generated previously [48] and bred with the ChAT‐Cre mice to conditionally knock out the *Gng13* gene in the ChAT‐expressing cells of the Gng13‐cKO mutant mice. Krt5‐CreER mouse line [49], a gift from Dr. Ying Xi, was crossed with Ai14 mice to generate Krt5‐CreER:Ai14 mice for cell lineage tracing. Mice were bred to more than 98% of C57BL/6 genetic background and maintained under a 12‐h light/dark cycle with access to water and food ad libitum in the Laboratory Animal Center of Zhejiang University. Progeny was genotyped by PCR, and both male and female mice at 6–8 weeks old were used in the experiments. Mice were euthanized using CO_2_ inhalation at 40% flow rate of chamber volume per minute. For H1N1 infection and olfactory tissue collection, mice were anesthetized with pentobarbital sodium administered intraperitoneally at 50 mg/kg bodyweight. Studies involving animals were approved by the Zhejiang University Institutional Animal Care and Use Committee, and performed following the NIH ‘Guidelines for the Care and Use of Laboratory Animals’.

### 2.3 Animal Models of Viral Infection

Mice between 6 and 8 weeks old were anesthetized with pentobarbital sodium (Sigma, P3761) at 50 mg/kg bodyweight, and then intranasally administered with 120 pfu of influenza virus A/Puerto Rico/8/1934 in 25 μL PBS on 0 days post infection (dpi). The mice were checked and weighed every day and euthanized for analysis at time points of 0, 5, 10 and 15 dpi. PBS‐treated, uninfected WT control mice were included at each time point.

For cell lineage tracing experiments, mice were treated as described previously with some modifications [49]. Briefly, Krt5‐CreER:Ai14 mice were intraperitoneally administered 5 doses of 200 mg/kg body weight tamoxifen (Sigma) dissolved in corn oil 3, 4, 5, 6 and 7 days before H1N1 infection. On 15 dpi, the mice were sacrificed, and their olfactory epithelia were dissected out for immunohistochemical analysis.

### 2.4 X‐Gal Staining

X‐gal staining experiment was performed as previously described [44]. Trpm5‐lacZ mice with or without H1N1 infection were fully anesthetized with pentobarbital sodium and transcardially perfused with 4% paraformaldehyde (PFA) in PBS. Olfactory turbinates were dissected out in ice‐cold PBS under a stereo microscope and post‐fixed in 4% PFA overnight, followed by cryoprotection in 30% sucrose in PBS at 4°C overnight and decalcification in 250 mM EDTA in PBS for 3 days. Then the olfactory tissues were embedded in Tissue‐Tek Optimal Cutting Temperature (O.C.T.) medium (Sakura, 4583) and sliced coronally into sections of 12 μm thickness using Cryostat Microm (Thermo Scientific, HM525). The olfactory epithelium slices were washed once in the permeabilization buffer (2 mM MgCl_2_, 0.02% NP40 in PBS) for 10 min and twice in the washing buffer (2 mM MgCl_2_ in PBS) for 10 min and then rinsed in the detergent solution (2 mM MgCl_2_, 0.02% NP40, 0.01% deoxycholate in PBS) for 10 min. The sections were incubated in the β‐galactosidase substrate (2 mM MgCl_2_, 0.02% NP40, 0.01% deoxycholate, 5 mM K_3_Fe(CN)_6_, 5 mM K_4_[Fe(CN)_6_]·3H_2_O, 1 mg/mL X‐gal in PBS) in the dark for 5 h at room temperature. The stained slices were washed twice in PBS and imaged using the Nikon AZ100 microscope.

### 2.5 Immunohistochemistry

Olfactory epithelium was harvested and processed as described previously [50]. Briefly, mice were fully anesthetized with pentobarbital sodium and transcardially perfused with 4% PFA in PBS. Olfactory turbinates were dissected out and post‐fixed in 4% PFA overnight, followed by cryoprotection in 30% sucrose in PBS at 4°C overnight and decalcification in 250 mM EDTA in PBS for 3 days. Then the olfactory tissues were embedded in the O.C.T. medium and sliced coronally into sections of 12 μm thickness using Cryostat Microm. For all immunostaining, antigen retrieval was performed by microwaving the tissue sections in 10 mM Na Citrate/0.05% Tween 20, pH 6.0 for 20 min. Then the tissue sections were blocked in the blocking buffer (3% BSA, 0.3% Triton X‐100, 2% donkey serum and 0.1% sodium azide in PBS) for 1 h at RT, followed by incubation at 4°C overnight with primary antibodies diluted in the blocking buffer. Primary antibodies used were as follows: rabbit anti‐Dclk1 (1:500, Abcam, ab31704), rabbit anti‐Gα‐gustducin (1:100, Santa Cruz Biotechnology, sc‐395), rabbit anti‐neurogranin (1:500, Sigma, AB5620), rabbit anti‐COX‐1 (1:200, Proteintech, 13393‐1‐AP), mouse anti‐IL‐25 (1:100, R&D, MAB1258), rabbit anti‐Lrmp (1:200, Biorbyt, orb166443), rabbit‐anti‐H1N1 Influenza A virus nucleocapsid protein (1:200, Abcam, ab104870), rat anti‐Ki67 (1:200, Thermo Fisher, 14569882), rabbit anti‐SOX2 (1:200, Abcam, ab97959), goat anti‐ICAM1 (1:200, R&D, AF796), rabbit anti‐CD45 (1:500, Abcam, ab10558), rat anti‐F4/80 (1:200, Bio‐Rad, MCA497GA), rat anti‐CD3 (1:200, Thermo Fisher, 14003282), rabbit anti‐GATA3 (1:200, Abcam, ab199428), mouse anti‐caspase‐1 (1:200, Santa Cruz Biotechnology, sc‐56036), rabbit anti‐Gsdmd (1:200, Abcam, ab219800), rabbit‐anti‐cleaved caspase‐3 (1:200, Abcam, ab32042), rabbit anti‐Ascl1 (1:50, Abcam, ab211327), and chicken anti‐GFP polyclonal antibody (1:100, ab13970, Abcam). The slices were washed and incubated with the secondary antibodies: donkey anti‐rabbit IgG H&L Alexa Fluor 488 (1:500, Abcam, ab150073), goat anti‐rabbit IgG H&L Alexa Fluor 647 (1:500, Abcam, ab150079), donkey anti‐goat IgG H&L Alexa Fluor 488 (1:500, Abcam, ab150129), donkey anti‐rat IgG H&L Alexa Fluor 488 (1:500, Thermo Fisher, a‐21208), goat anti‐chicken IgY H&L Alexa Fluor 488 (1:500, ab150169, Abcam), donkey anti‐mouse IgG (H + L) Highly Cross‐Adsorbed Secondary Antibody Alexa Fluor 488 (1:500, Thermo Fisher, a‐21202), donkey anti‐rat IgG (H + L) Highly Cross‐Adsorbed Secondary Antibody Alexa Fluor Plus 647 (1:500, Thermo Fisher, a48272) for 1.5 h at RT. Fluorescent images were captured using an FV3000 laser scanning confocal microscope (Olympus).

### 2.6 Quantitative Reverse Transcription‐PCR

Olfactory turbinates were taken out with a pair of tweezers after the nasal cavity was cut open longitudinally along the midline. The tissues were washed in PBS and lysed to isolate total RNAs using the TaKaRa MiniBEST Universal RNA Extraction Kit (TaKaRa, 9767), which were reverse transcribed into cDNAs using the RevertAid First Strand cDNA Synthesis Kit (Thermo Fisher Scientific, K1622). qPCR reactions were set up using iQ SYBR Green Supermix (Bio‐rad, 1708884) and run on the CFX Connect Real‐Time System. Relative expressions were calculated and normalized to the expression of the internal control gene *Actb*. The primer pairs used in these qPCR reactions are listed in Table S1.

### 2.7 Olfactory Organoid Culture and Treatment

Olfactory epithelial organoids were cultured as previously described with some modifications [51]. Briefly, the olfactory epithelium was dissected out from the ChAT‐Cre: Ai9 mice, washed repeatedly with ice‐cold PBS and then minced and incubated with 0.25% trypsin–EDTA plus 70 U/mL DNase I at 37°C for 20 min. After trituration, cells were filtered through a 40‐μm cell strainer (Falcon, 352340), and individual cells in the filtrate were collected by centrifugation (500 × g, 5 min) at 4°C and resuspended in growth factor reduced Matrigel (Corning, 356231) and plated in preheated 24‐well plates. Once the Matrigel was solidified, the culture medium [DMEM/F12 medium (Thermo Fisher, 10565018) supplemented with 50 ng/mL Wnt3a (Novoprotein, C18K), 50 ng/mL EGF (R&D, 236‐EG), 100 ng/mL Noggin (PeproTech, 250‐38), 200 ng/mL R‐spondin 1 (R&D, 4645‐RS‐025), as well as antibiotic‐antimycotic (Thermo Fisher, 15240062), 1% GlutaMax (Thermo Fisher, 35050061), 10 mM HEPES (Thermo Fisher, 15630080), N2 (Thermo Fisher, 17502048), B27 (Thermo Fisher, 17504044), 10 μM Y‐27632 (MCE, HY‐10071), 20 ng/mL FGF (R&D, 233‐FB)] was added to each well.

After grown for 7 days in the above medium, the olfactory organoids were treated with cytokines for 7 more days: 200 ng/mL mouse IL‐4 protein (Novoprotein, CK74), 200 ng/mL mouse IL‐13 protein (Novoprotein, CH18), 200 ng/mL mouse IL‐1β protein (Novoprotein, C042), 200 ng/mL human IL‐25 protein (Novoprotein, C792), 200 ng/mL mouse TNF‐α protein (Novoprotein, CF09), 50 ng/mL mouse IFN‐γ protein (Novoprotein, C746) while the control organoids were cultured for 14 days without any cytokines. The bright field and fluorescent images were captured using an FV3000 laser scanning confocal microscope for 80–100 μm Z‐stack. For immunohistochemistry, the chemokine‐treated or ‐untreated organoids were released from Matrigel, washed and fixed in 4% PFA for 15 min, followed by cryo‐protection in 30% sucrose overnight. The organoids were then embedded in the O.C.T. medium and sliced into 12 μm‐thick sections. For qRT‐PCR, the released organoids from Matrigel were washed in ice‐cold PBS, and then used for total RNA isolation using TaKaRa MiniBEST Universal RNA Extraction Kit. The total RNA was reverse transcribed into cDNAs, which were used for qPCR reactions as described above. The qPCR primers are listed in Table S1.

### 2.8 scRNA‐Seq Data Reanalysis

The single‐cell RNA sequencing dataset from GSE245074 [4] was reanalyzed to investigate the expression of specific genes in nasal cell populations at homeostasis. The Seurat package (v4.0) was employed to extract and process the relevant raw data. To visualize the heterogeneity of tuft cells and their potential influence on olfactory stem cell regulation, *t*‐SNE plots were constructed.

### 2.9 Statistical Analysis

Experimental data were obtained from three or more mice or other biological replicates per group. Data normality was assessed using the Shapiro–Wilk test before parametric analysis. For comparisons among multiple groups within a single variable, one‐way ANOVA was used. For comparisons involving two independent variables, such as genotype and time post‐infection, two‐way ANOVA was performed. Post hoc multiple comparisons were performed using Tukey's tests. For in vivo experiments, each biological replicate (*n*) represents an individual animal. For immunostaining analysis, images from six randomly selected areas of each animal's tissue sections were post hoc processed with ImageJ, and immuno‐labelled cells were counted per unit length of olfactory neuroepithelium as described previously [52]. For organoid experiments, *n* represents the number of independent experiments. Data were analyzed using the GraphPad Prism 9 software and are presented as mean ± SD. *p* values < 0.05 were considered statistically significant.

## 3 Results

### 3.1 Olfactory Tuft Cells Express Gustatory Signalling Proteins and Other Tuft Cell Markers Except Dclk1

**FIGURE 1:**
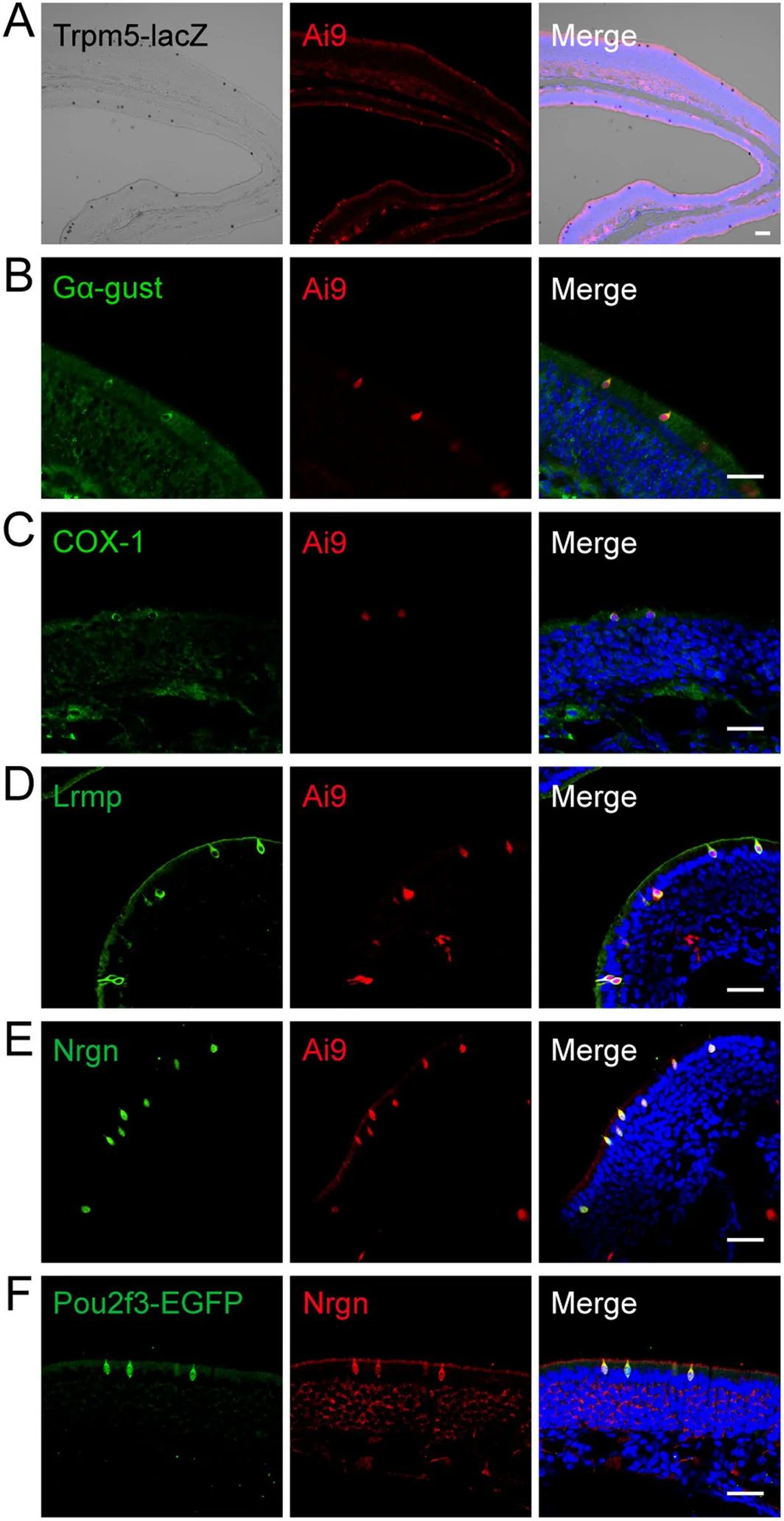
Olfactory tuft cells express gustatory signalling proteins and other marker proteins. (A) The olfactory epithelial tissue sections from the ChAT‐Cre:Ai9:Trpm5‐lacZ^+/−^ mice were stained with X‐gal (blue). Nearly all ChAT‐Cre:Ai9 (tdTomato)‐positive cells (Ai9, red) were labelled with X‐gal staining (blue). (B–E) Olfactory epithelial tissue sections from the ChAT‐Cre:Ai9 mice were stained with antibodies against Gα‐gustducin (green) (B), COX‐1 (green) (C), Lrmp (D) and neurogranin (Nrgn) (E). (F) Olfactory epithelial tissue sections from the Pou2f3‐CreERT2‐IRES‐EGFP mice were stained with the anti‐neurogranin (Nrgn) antibody; and DAPI (blue) for nuclei. All these marker proteins were colocalized with Ai9 or EGFP to olfactory tuft cells. Scale bar: 50 μm.

Previous studies have identified two types of olfactory microvillous cells, MV1 and MV2 [4, 27]. To verify that MV1 cells are indeed olfactory tuft cells, we generated ChAT‐Cre:Ai9:Trpm5‐lacZ^+/−^ mice by crossing ChAT‐Cre:Ai9 mice with the Trpm5‐lacZ knock‐in line, in which the lacZ‐encoded β‐galactosidase is faithfully expressed in the *Trpm5*‐expressing cells. X‐gal staining showed that 88.3% of Trpm5‐lacZ^+^ microvillous cells were ChAT‐Cre:Ai9^+^ whereas 96.7% of ChAT‐Cre:Ai9^+^ cells in the olfactory epithelium coexpressed Trpm5‐lacZ (Figures 1A and S1A), supporting that both markers reliably identify olfactory tuft cells. Immunostaining with antibodies against Gα‐gustducin—a Gα subunit essential for sweet, bitter and umami taste sensation, cyclooxygenase I (COX‐1)—a key enzyme for prostaglandin biosynthesis, and a pan‐tuft cell marker, lymphoid‐restricted membrane protein (Lrmp), as well as an olfactory tuft cell‐selective marker, neurogranin [4] revealed that 96.1% of Gα‐gustducin^+^, 99.9% of COX‐1^+^, 99.4% of Lrmp^+^ and 98.9% of neurogranin^+^ cells were also ChAT‐Cre:Ai9^+^ while 87.7%, 95%, 94.7% and 96% of ChAT‐Cre:Ai9^+^ cells coexpressed Gα‐gustducin, COX‐1, Lrmp and neurogranin, respectively (Figures 1B–E and S1B–E). Pou2f3 is an essential transcription factor required for tuft cell development. Immunostaining of the olfactory epithelium from the Pou2f3‐CreERT2‐IRES‐EGFP knockin mice showed that 99.6% of neurogranin^+^ cells were also Pou2f3^+^ whereas 96.1% of Pou2f3^+^ cells also expressed neurogranin (Figures 1F and S1F). To determine whether tuft cells were retained in olfactory epithelial organoids and expressed the tuft cell markers as well as the output signalling molecule, interleukin 25 (IL‐25) [35], we generated these organoids from the ChAT‐Cre:Ai9 mice and performed immunofluorescence on the organoid sections with antibodies against Gα‐gustducin and IL‐25; and the results showed that ChAT‐Cre:Ai9^+^ tuft cells were indeed present in the olfactory epithelial organoids, coexpressing Gα‐gustducin and IL‐25 (Figure S2).

Reanalysis of previously published single‐cell RNA sequencing data [4] showed that olfactory tuft cells express a suite of canonical gustatory transduction and immune‐related genes, including *Gnat3*, *Gng13*, *Plcb2*, *Itpr2*, *Itpr3*, *Trpm5*, *Il25*, *Ptgs1*, *Il13ra1*, *Il4ra* and *Il17rb*, encoding Gα‐gustducin, Gγ13, phospholipase Cβ2, inositol 1,4,5‐triphosphate receptor type 2 (IP3R2), IP3R3, Trpm5, IL‐25, COX‐1, IL‐13 receptor α1, IL‐4 receptor α and IL‐17 receptor B, respectively (Figure S3). These findings demonstrate that MV1 cells are indeed olfactory tuft cells, characterized by expression of Trpm5, ChAT, COX‐1 and key cytokines and their receptors. Notably, olfactory tuft cells do not express Dclk1, a widely used tuft cell marker, which, however, was found in the cells of Bowman's glands (Figure S4) [4].

Collectively, these results show that olfactory tuft cells express a unique combination of chemosensory and immunomodulatory genes, suggesting their specialized roles in olfactory epithelial immunity and sensory function.

### 3.2 Loss of Gγ13 and Trpm5 Heightens Baseline Inflammation and Amplifies Immune Cell Infiltration Following H1N1 Infection in the Olfactory Mucosa

**FIGURE 2:**
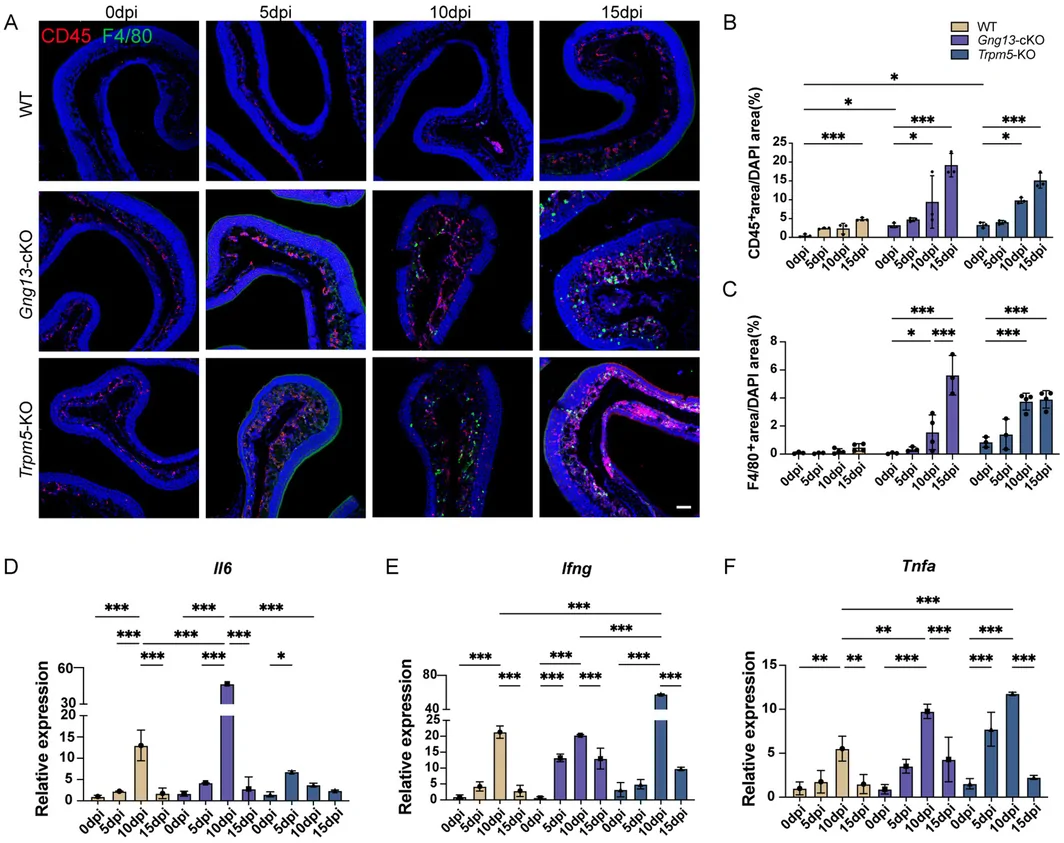
Ablation of Gγ13 and Trpm5 increases type 1‐associated inflammation that is exacerbated by H1N1 infection. (A) Representative images of the olfactory epithelium tissue sections of WT, Gng13‐cKO and Trpm5‐KO mice before or after H1N1 infection at 0, 5, 10 and 15 dpi that were stained with antibodies against CD45 (red) and F4/80 (green) and DAPI (blue). Scale bar: 50 μm. (B, C) Statistical analysis of the areas of CD45^+^ immune cells (B) and F4/80^+^ macrophage cells (C) over the DAPI‐stained areas. Each dot represents a mouse; data are presented as means ± SD; Two‐way ANOVA were performed. (D–F) qRT‐PCR analysis of the pro‐inflammatory cytokine *Il6* (D), type 1‐associated cytokines *Ifng* (E) and *Tnfa* (F) gene expression in WT, Gng13‐cKO, and Trpm5‐KO olfactory mucosa before and after H1N1 infection. Data are presented as means ± SD (*n* = 3); Two‐way ANOVA were performed. *p < 0.05, **p < 0.01, ***p < 0.001.

To investigate the role of gustatory signalling proteins in olfactory mucosal immunity, we generated *Gng13*‐conditional knockout (Gng13‐cKO) mice by crossing Gng13^flox/flox^ mice with ChAT‐Cre mice and used a previously established Trpm5‐KO line [39]. Under basal conditions (0 days post‐infection, dpi), immunostaining with antibodies against a pan‐immune cell marker, CD45, and a macrophage marker, F4/80, revealed minimal immune cell presence in wild‐type (WT) olfactory tissues (0.5% CD45^+^ area/DAPI area) (Figure 2A,B). In contrast, Gng13‐cKO and Trpm5‐KO mice exhibited significantly elevated CD45^+^ immune cell infiltration (3.19% and 3.26%, respectively). However, F4/80^+^ macrophages remained low across all genotypes, and the differences were not statistically significant (0.08%, 0.05% and 0.85% in WT, Gng13‐cKO and Trpm5‐KO, respectively; Figure 2A,C).

To assess susceptibility to viral infection, we exposed mice to H1N1 influenza virus [53]. Immunostaining for viral nucleoprotein (NP) revealed broad infection across the olfactory epithelium under a high‐dose challenge (Figure S5A), while a low‐dose infection localized NP expression primarily to sustentacular cells (Figure S5B). Notably, co‐localization of NP and Ai9 confirmed that tuft cells were directly infected (Figure S5C), a finding supported by similar infections observed in olfactory organoids (Figure S5D). Following sublethal H1N1 inoculation, CD45^+^ cell numbers increased in all genotypes (Figures 2A,B and S6). In WT mice, this increase was statistically significant only at 15 dpi (4.84% vs. 0.47% at 0 dpi). In contrast, both Gng13‐cKO and Trpm5‐KO mice showed more robust responses, with CD45^+^ cells significantly increased at both 10 and 15 dpi (Figures 2A,B and S6). F4/80^+^ macrophages displayed distinct temporal dynamics (Figures 2A,C and S6). WT tissues showed no significant changes, while Gng13‐cKO and Trpm5‐KO exhibited significant increases at 15 and 10 dpi, respectively. To assess cytokine responses, we performed qRT‐PCR for the proinflammatory cytokine *Il6*, and the two type 1‐associated cytokines: *Ifng* and *Tnfa* (Figure 2D–F). *Il6* and both type 1‐associated cytokine genes were significantly upregulated after infection, but their kinetics differed. *Il6* peaked at 5 dpi in Trpm5‐KO mice, at 10 dpi in both WT and Gng13‐cKO mice. Gng13‐cKO tissues showed the highest *Il6* levels, while Trpm5‐KO showed the lowest, with all groups returning to baseline by 15 dpi (Figure 2D). *Ifng* expression peaked at 10 dpi across all genotypes, with Trpm5‐KO tissues showing the highest level. Notably, Gng13‐cKO mice exhibited an earlier response, with significant elevation by 5 dpi and a peak at 10 dpi before declining (Figure 2E). *Tnfa* followed similar trends in WT and Gng13‐cKO, peaking at 10 dpi, while in Trpm5‐KO mice, *Tnfa* spiked at 5 dpi, remained elevated through 10 dpi, then declined. Overall, *Tnfa* expression in Trpm5‐KO was comparable to Gng13‐cKO but exceeded WT (Figure 2F).

**FIGURE 3:**
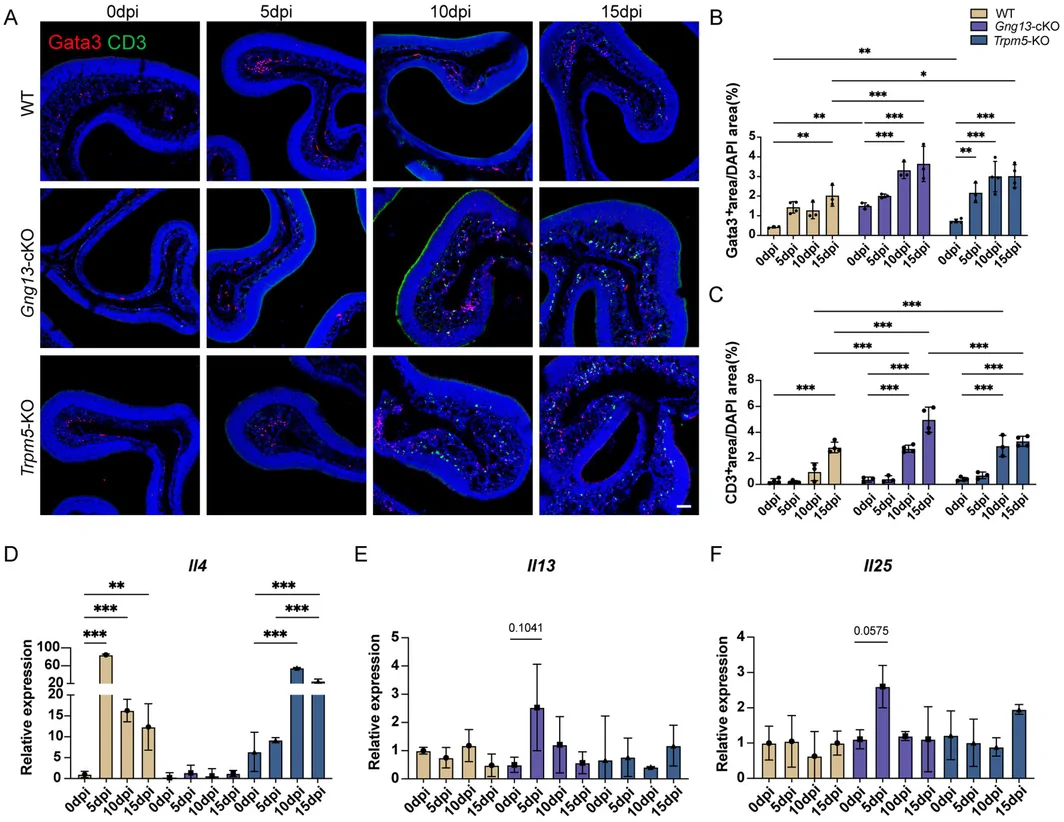
Ablation of Gγ13 and Trpm5 and H1N1 infection evoked more immune cell infiltration. (A) Representative images of olfactory epithelium tissue sections of WT, Gng13‐cKO, Trpm5‐KO mice at 0, 5, 10 and 15 dpi following H1N1 infection that were stained with the antibodies against GATA3 (red) and CD3 (green), and DAPI (blue) for nuclei. Scale bar: 50 μm. (B, C) Statistical analysis of the areas of GATA3^+^ ILC2s (B) and CD3^+^ T cells (C) over their corresponding DAPI‐stained areas. Each dot represents a mouse; data are presented as means ± SD; Two‐way ANOVA were performed. (D–F) qRT‐PCR analysis of type 2‐associated cytokines *Il4* (D), *Il13* (E) and *Il25* (F) gene expression in WT, Gng13‐cKO, Trpm5‐KO olfactory mucosa before and after H1N1 infection. Data are presented as means ± SD (*n* = 3); Two‐way ANOVA were performed. *p < 0.05, **p < 0.01, ***p < 0.001.

To further characterize immune cell infiltration, we quantified GATA3^+^ Group 2 innate lymphoid cells (ILC2s) and CD3^+^ T cells (Figures 3 and S7). At baseline, Gng13‐cKO and Trpm5‐KO mice harboured significantly more GATA3^+^ ILC2s (1.50% and 0.73%) than WT (0.43%) (Figure 3A,B). Following infection, Trpm5‐KO mice showed an early increase at 5 dpi; Gng13‐cKO followed at 10 dpi, and WT lagged behind, peaking at 15 dpi. At 10 and 15 dpi, both mutants had significantly more GATA3^+^ cells than WT. CD3^+^ T cells showed patterns similar to F4/80^+^ macrophages (Figure 3C). Baseline differences were not statistically significant, but both mutants exhibited strong increases at 10 dpi. WT mice showed a delayed T cell response, with significance reached only at 15 dpi. Besides, we also detected the mRNA expression of type 2 immune‐associated cytokines. Unlike the type 1‐associated cytokines, type 2‐associated cytokine genes displayed different expression profiles. *Il4* was most strongly induced in WT mice, peaking at 5 dpi (~80‐fold increase), followed by a gradual decline. Trpm5‐KO showed delayed induction at 10 dpi, while Gng13‐cKO showed no response at all (Figure 3D). Neither *Il13* nor *Il25* showed significant changes in any genotype after infection (Figure 3E,F).

In summary, loss of Gγ13 or Trpm5 heightened basal infiltration of CD45^+^ immune cells and GATA3^+^ ILC2s. H1N1 infection further amplified immune infiltration, with Gng13‐cKO showing stronger recruitment of macrophages and T cells. Cytokine gene expression analysis revealed genotype‐specific effects: Gng13‐cKO enhanced *Il6* expression but suppressed *Il4* expression, while Trpm5‐KO accelerated *Tnfa* and *Ifng* expression but dampened *Il6* expression. These findings indicate that tuft cell–mediated gustatory signalling contributes to both baseline immune homeostasis and virus‐induced immune responses in the olfactory mucosa.

### 3.3 Olfactory Tuft Cell Signalling Deficiencies Heighten Apoptosis and Pyroptosis, Particularly Following H1N1 Infection

To determine whether tuft cell signalling impacts epithelial cell survival, we assessed apoptosis in the olfactory tissues of WT, Gng13‐cKO and Trpm5‐KO mice via cleaved caspase‐3 immunostaining. Under basal conditions (0 dpi), cleaved caspase‐3^+^ apoptotic cells were sparse in WT tissues (0.45% cleaved caspase‐3^+^ area/DAPI; Figure S8). Gng13‐cKO mice exhibited significantly more cleaved caspase‐3^+^ cells than WT, while Trpm5‐KO mice showed no difference at baseline. Upon H1N1 infection, the apoptotic cell numbers remained unchanged in WT but significantly increased in both mutant mouse lines by 10 dpi.

**FIGURE 4:**
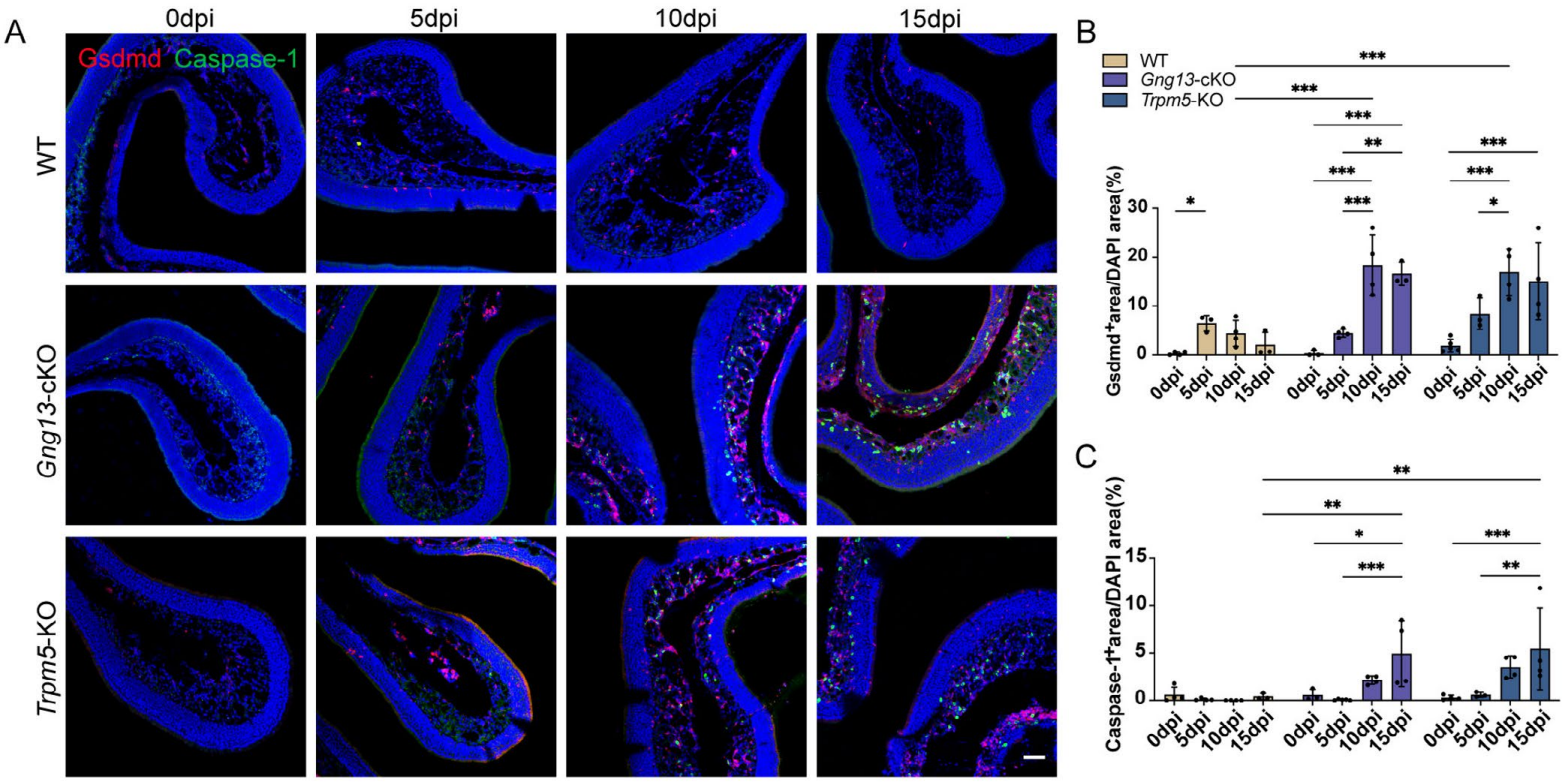
Ablation of Gγ13 and Trpm5 exacerbates H1N1 infection‐induced pyroptosis in the olfactory tissue. (A) Representative images of WT, Gng13‐cKO, Trpm5‐KO olfactory epithelial tissue sections at 0, 5, 10 and 15 dpi stained with antibodies against Gsdmd (red) and caspase‐1 (green) along with DAPI to visualize the nuclei (blue). Scale bar: 50 μm. (B, C) Statistical analysis of the areas of Gsdmd^+^ (B) and caspase‐1^+^ pyroptotic cells (C) over the areas of DAPI, respectively. Each dot represents a mouse; data are presented as means ± SD. Two‐way ANOVA were performed. *p < 0.05, **p < 0.01, ***p < 0.001.

To examine pyroptosis, we performed immunostaining for gasdermin D (Gsdmd) and caspase‐1, the two key markers of inflammasome‐driven cell death (Figures 4 and S9). At 0 dpi, Gsdmd^+^ and caspase‐1^+^ cells were rare across all genotypes. Following H1N1 infection, WT mice exhibited a rapid but transient increase in Gsdmd^+^ cells, peaking at 5 dpi and declining thereafter. In contrast, Gng13‐cKO and Trpm5‐KO mice showed a delayed response, with the Gsdmd^+^ cell density peaking at 10 dpi. Importantly, the mutant mice displayed substantially higher Gsdmd^+^ cell levels at their respective peaks compared to WT (Figure 4B). Caspase‐1^+^ cell density remained unchanged in WT mice post‐infection. However, both Gng13‐cKO and Trpm5‐KO mice exhibited a significant rise at 15 dpi, with levels exceeding those in WT (Figure 4C), suggesting a prolonged and heightened inflammasome activation in the absence of functional tuft cell signalling.

To further investigate pyroptosis‐related gene expression, we quantified *Nlrp3* and *Il1b* gene expression via qRT‐PCR (Figure S10). *Nlrp3* expression remained stable in WT and Gng13‐cKO mice after infection. In contrast, Trpm5‐KO mice showed a transient but significant increase at 5 dpi, which declined by 10 and 15 dpi. For *Il1b*, expression was modestly increased at 10 dpi in WT and Gng13‐cKO mice, but Trpm5‐KO mice exhibited an earlier and more robust upregulation. Across all time points, *Il1b* levels were the highest in Trpm5‐KO, although all genotypes returned to near‐basal expression by 15 dpi.

In summary, Gng13‐cKO and Trpm5‐KO mice exhibit increased epithelial apoptosis and pyroptosis compared to WT in response to H1N1 infection. The Trpm5 deficiency results in heightened inflammasome activation, marked by the elevated Gsdmd, caspase‐1, and *Il1b* expression. Conversely, WT olfactory tissues remain largely resistant to apoptosis and display a more regulated pyroptotic response.

### 3.4 Loss of Gγ13 or Trpm5 Increases Olfactory Tuft Cell Numbers, Further Augmented by H1N1 Infection

**FIGURE 5:**
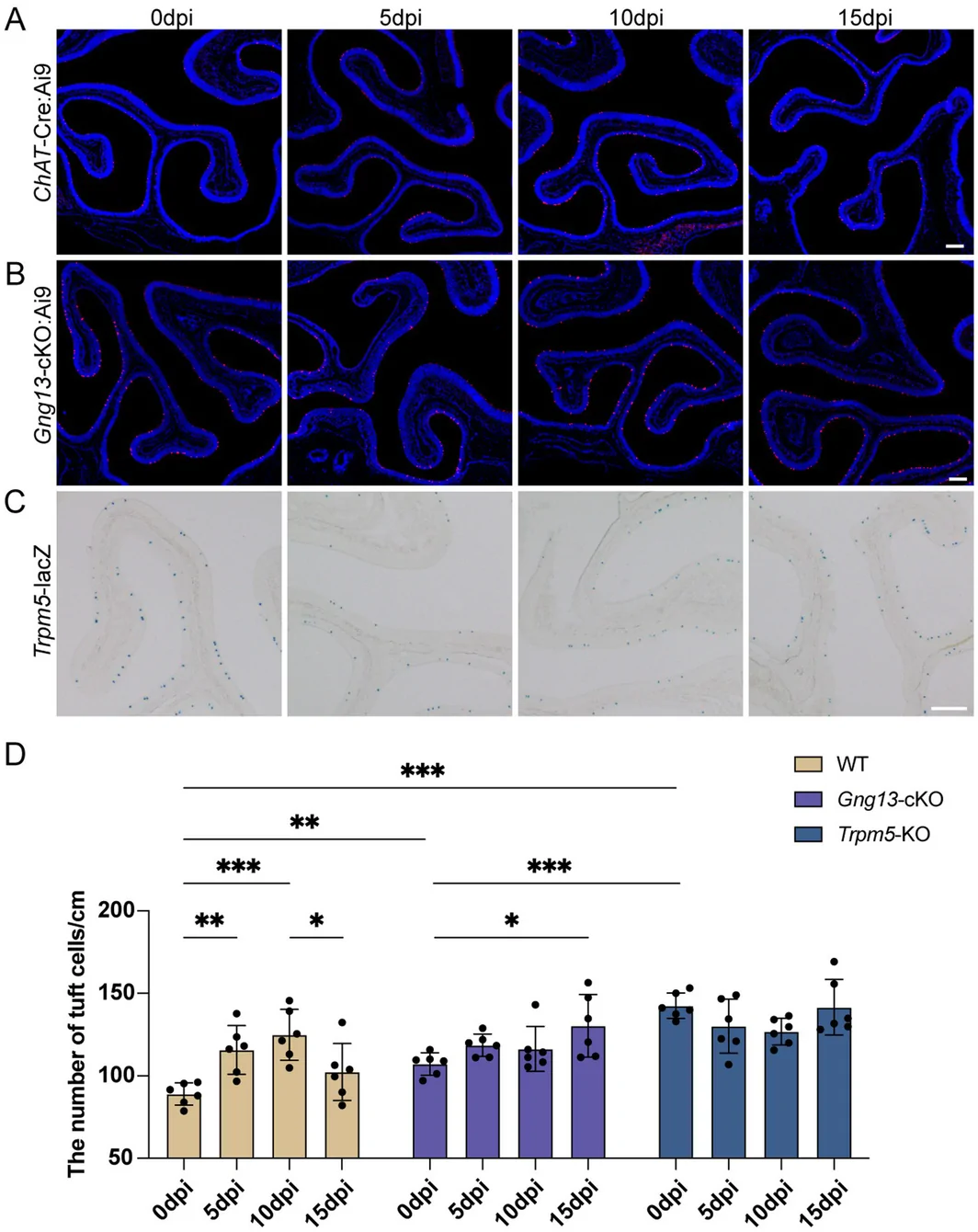
Ablation of Gγ13 or Trpm5 and H1N1 infection induce olfactory tuft cell hyperplasia. (A, B) Representative images of olfactory epithelium sections from H1N1‐uninfected and ‐infected ChAT‐Cre: Ai9 mice (A) and ChAT‐Cre: Ai9: Gng13flox/flox mice (Gng13‐cKO:Ai9) (B) at 0, 5, 10 and 15 dpi. The tissues were stained with DAPI (blue). (C) Representative images of olfactory epithelium sections from H1N1‐uninfected and infected Trpm5‐KO (Trpm5‐lacZ) mice with X‐gal staining at 0, 5, 10 and 15 dpi. Scale bar: 50 μm. (D) Statistical analysis of the number of ChAT‐Ai9+ or Trpm5‐lacZ+/+ olfactory tuft cells per cm of the olfactory epithelium of ChAT‐Cre:Ai9, Gng13‐cKO: Ai9 and Trpm5‐lacZ mice. Each dot represents a mouse; data are shown as means ± SD. Two‐way ANOVA were performed. *p < 0.05, **p < 0.01, ***p < 0.001.

Given the enhanced immune cell infiltration, elevated cytokine expression, and increased cell death in the olfactory mucosa of Gng13‐cKO and Trpm5‐KO mice, we next asked whether the abundance of olfactory tuft cells was altered in these mutants. To visualize and quantify tuft cells, we employed lineage‐labelling strategies specific to each genotype. In WT controls, we used ChAT‐Cre:Ai9 mice, which express tdTomato in ChAT‐expressing tuft cells. Gng13‐cKO:Ai9 mice were generated by crossing ChAT‐Cre:Ai9 mice with Gng13^flox/flox^ mice, resulting in the *Gng13* deletion and simultaneous tuft cell labelling. Trpm5‐KO mice contain a lacZ cassette inserted into the Trpm5 locus, allowing tuft cell visualization by X gal staining (Figure 5A–C). Quantification revealed that, under basal conditions (0 dpi), both Gng13‐cKO and Trpm5‐KO mice exhibited significantly more tuft cells than WT mice (107.13 ± 6.78 and 142.49 ± 7.70 vs. 88.92 ± 6.75 cells/cm of olfactory epithelium, respectively; Figure 5D). Notably, Trpm5‐KO mice had more tuft cells than Gng13‐cKO, indicating that the extent of hyperplasia varies with the disrupted signalling components. Upon H1N1 infection, WT mice showed a transient expansion of tuft cells, peaking at 10 dpi followed by a decline at 15 dpi. In contrast, Gng13‐cKO mice displayed a slow increase in tuft cell numbers over the time course, suggesting an impaired regenerative response. Trpm5‐KO mice, however, did not show any further significant increase from their elevated basal level following H1N1 infection (Figure 5D).

These results indicate that the nullification of *Gng13* or *Trpm5* promotes tuft cell hyperplasia under homeostatic conditions. Moreover, the dynamics of tuft cell proliferation during H1N1 infection diverge across genotypes: WT mice mount a transient, infection‐induced tuft cell expansion, while Gng13‐cKO mice exhibit a blunted response, and Trpm5‐KO mice do not show any further increase.

### 3.5 Tuft Cell Hyperplasia in Gγ13‐andTrpm5‐Deficient Mice Arises From Enhanced HBCProliferation and Differentiation, Further Amplified by H1N1 Infection

The hyperplasia of tuft cells observed in both Gng13‐cKO and Trpm5‐KO mice, both under basal conditions and following H1N1 infection, prompted us to examine whether the reserved stem cells in the olfactory epithelium, HBCs, were activated and contributed to tuft cell expansion. To this end, we carried out cell lineage tracing experiments using the Krt5‐CreER:Ai14 mice. These mice were intraperitoneally administered with 5 doses of tamoxifen, followed by H1N1 infection. Immunostaining on the olfactory epithelial tissue sections with the anti‐neurogranin antibody at 0 and 15 dpi showed that while no Ai14^+^ neurogranin^+^ tuft cells were found at 0 dpi, a subset of neurogranin^+^ tuft cells were also Ai14^+^ at 15 dpi (Figure S11). Furthermore, these Ai14^+^ neurogranin^+^ tuft cells appeared in two patterns in the olfactory epithelia, that is, individually or together with a group of Ai14^+^ sustentacular cells. To verify that some of these tuft cells directly originated from HBCs instead of GBCs, we examined the olfactory epithelia of Pou2f3‐CreERT2‐IRES‐EGFP mice at 0 and 7 dpi following H1N1 infection. The results indicated that at 0 dpi, none of the EGFP^+^ cells expressed the GBC marker Ascl1 (Figure S12). However, at 7 dpi, a number of EGFP^+^ cells were found on the olfactory epithelium, some of which were EGFP/Ascl1 double positive while others were EGFP^+^Ascl1^−^, suggesting that the latter originated from HBCs.

**FIGURE 6:**
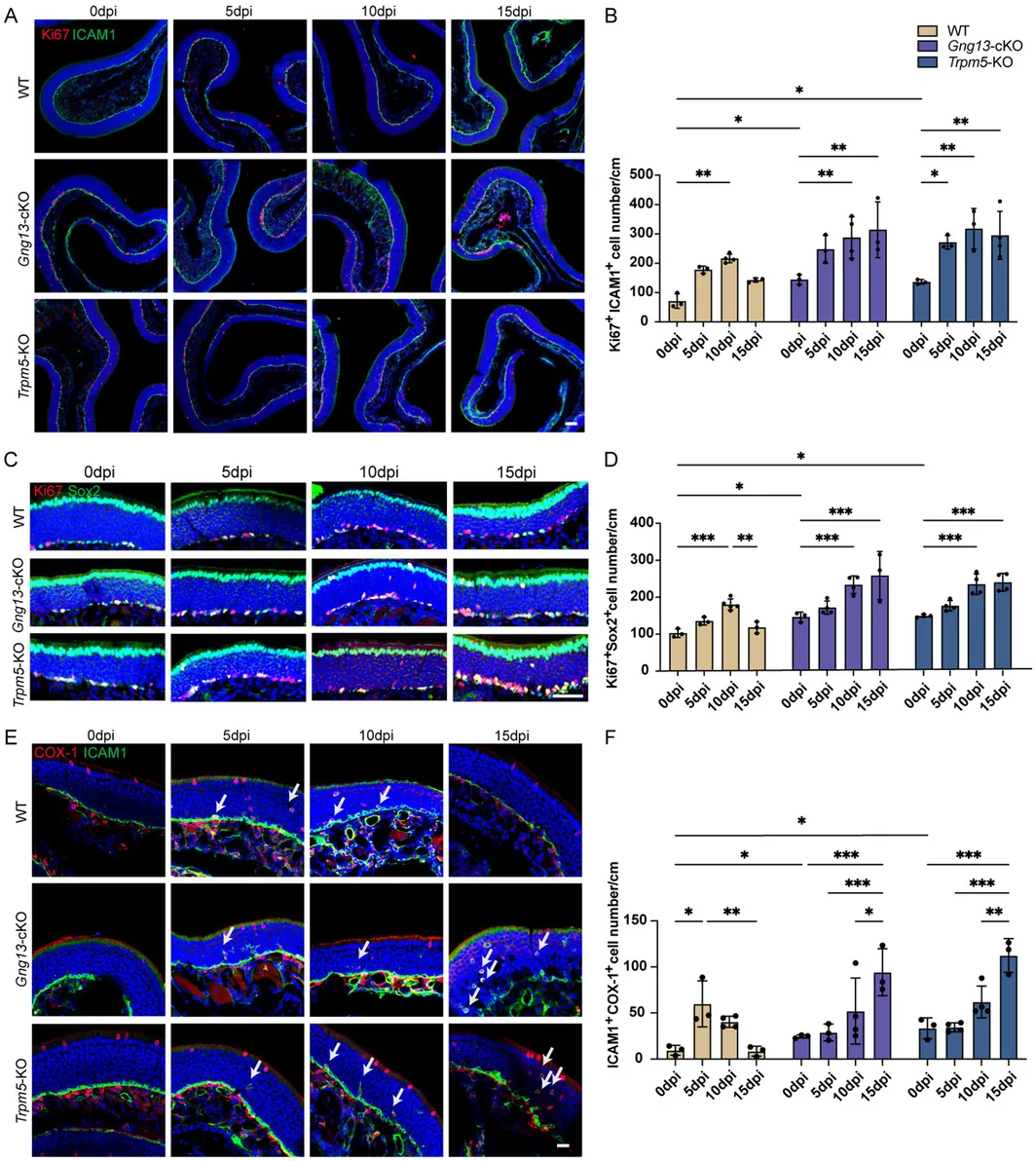
Genetic ablation of Gγ13 or Trpm5 enhances HBC proliferation and differentiation following H1N1 infection. (A, C, E) Representative images of olfactory epithelial sections from H1N1‐uninfected and infected WT, Gng13‐cKO and Trpm5‐KO mice at 0, 5, 10 and 15 dpi, immunostained for Ki67 (red) and ICAM1 (green) (A), Ki67 (red) and Sox2 (green) (C), the olfactory tuft cell marker COX‐1 (red) and the HBC marker ICAM1 (green) (E), with DAPI (blue) labelling nuclei. Arrows point to the COX‐1^+^ ICAM1^+^ cells. Scale bar, 50 μm. (B, D, F) Quantification of Ki67^+^ICAM1^+^ (B), Ki67^+^Sox2^+^ (D) and ICAM1^+^COX‐1^+^ (F) dual‐positive cells per centimetre (cm) of olfactory epithelium in WT, Gng13‐cKO, and Trpm5‐KO mice. Each dot represents one mouse; data are presented as means ± SD. Two‐way ANOVA was performed. *p < 0.05, **p < 0.01, ***p < 0.001.

To further assess the effect of the gustatory signalling on HBC‐derived tuft cell generation following H1N1 infection, we conducted immunohistochemical analyses on olfactory tissue sections from WT, Gng13‐cKO and Trpm5‐KO mice, using antibodies for the HBC marker ICAM1 and the proliferation marker Ki67 at 0, 5, 10 and 15 dpi. At 0 dpi, both Gng13‐cKO and Trpm5‐KO mice showed significantly more Ki67^+^ICAM1^+^ cells compared with WT (Figure 6A,B). Some of these cells were observed in the middle of the pseudostratified olfactory epithelial layer. Following H1N1 infection, the number of Ki67^+^ICAM1^+^ cells increased across all genotypes, peaking at 10 dpi in WT and Trpm5‐KO mice, and at 15 dpi in Gng13‐cKO mice (Figure 6B). However, the kinetics of the increase differed significantly among genotypes: Trpm5‐KO mice exhibited an earlier increase at 5 dpi, while WT and Gng13‐cKO mice showed significant increases only at 10 dpi. Notably, WT mice experienced a rapid decline by 15 dpi, while Gng13‐cKO and Trpm5‐KO mice displayed a more gradual decrease that did not reach statistical significance (Figure 6B). To validate these findings and reduce technical variability associated with co‐localization of two markers, we used an alternative approach by staining for SOX2, a well‐established nuclear marker of olfactory stem cells and sustentacular cells, in combination with Ki67. This dual staining confirmed the aforementioned findings and further highlighted the differences in HBC activation among these genotypes (Figure 6C,D). Next, we assessed whether proliferating olfactory stem cells contributed directly to tuft cell expansion by co‐staining for tuft cell markers. Double immunostaining for Ki67 and the mature tuft cell marker neurogranin showed no co‐localization across all genotypes (Figure S13). Also, the neurogranin^+^ cells were found to be mostly located closely to the apical edge of the epithelium, suggesting that neurogranin was expressed in mature tuft cells, and too late during tuft cell maturation to co‐localize with the proliferating marker Ki67.

We then tested another tuft cell marker, COX‐1, and the double immunostaining results for ICAM1 and COX‐1 revealed a significant presence of ICAM1^+^COX‐1^+^ cells in the basal and middle epithelial layers (Figure 6E). At 0 dpi, Gng13‐cKO and Trpm5‐KO mice showed significantly higher numbers of ICAM1^+^COX‐1^+^ cells than WT. Following H1N1 infection, WT mice exhibited a rapid increase in ICAM1^+^COX‐1^+^ cells, peaking at 5 dpi before declining. In contrast, Gng13‐cKO and Trpm5‐KO mice, which started with higher baseline levels, exhibited a more sustained increase in ICAM1^+^COX‐1^+^ cells, with elevated numbers persisting through 15 dpi (Figure 6F). Together, these data indicate that mutations in Gγ13 and Trpm5 resulted in enhanced HBC activation, leading to increased tuft cell proliferation and differentiation, which were further amplified during H1N1 infection. The identification of Krt5‐CreER:Ai14^+^ neurogranin^+^ cells, Pou2f3‐EGFP^+^ Ascl1^−^ cells and ICAM1^+^ COX‐1^+^ double‐positive cells supports the hypothesis that some HBCs directly differentiate into tuft cells, driving tuft cell hyperplasia in these mutant mice.

### 3.6 Cytokines Drive HBC Proliferation and Tuft Cell Differentiation in Olfactory Epithelial Organoids

**FIGURE 7:**
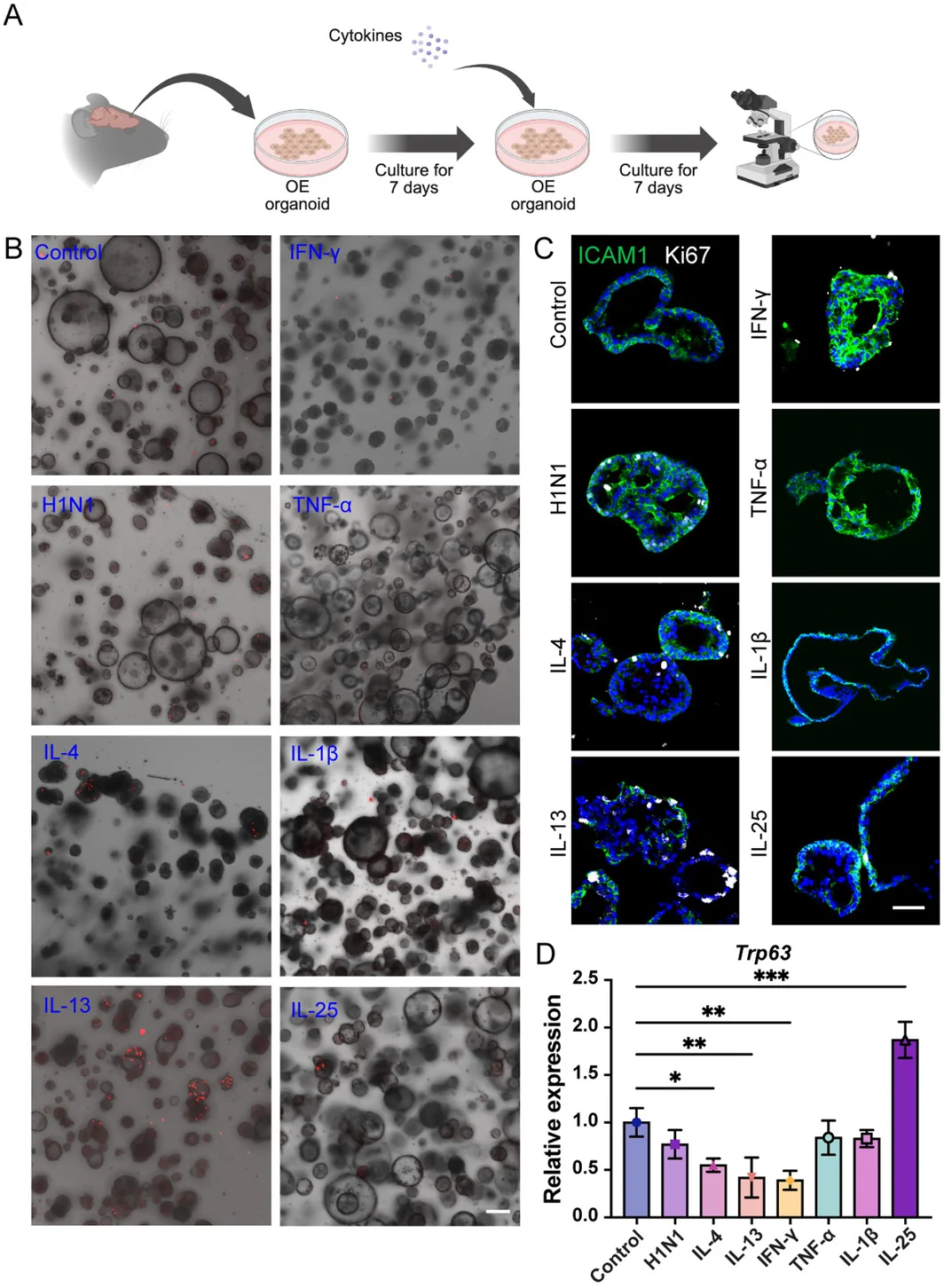
IL‐4 and IL‐13 activate HBCs, promoting a transitional state into tuft cells in the organoids. (A) Schematic illustration of the olfactory epithelial organoid culture and treatment. (B) Representative micrographs showing the morphology of untreated (control) and cytokines‐treated olfactory epithelial organoids derived from ChAT‐Cre:Ai9 mice, including H1N1, IL‐4, IL‐13, IFN‐γ, TNF‐α, IL‐1β and IL‐25. The organoids were cultured for 7 days to form spheroids and then treated with different cytokines for another 7 days. Bright field and red fluorescence images were taken sequentially and then merged using the software of the FV3000 system. Scale bar: 100 μm. (C) Representative images of the sections from the untreated (control), or H1N1‐, IL‐4‐, IL‐13‐, IFN‐γ‐, TNF‐α‐, IL‐1β‐ or IL‐25‐ treated olfactory epithelial organoids that were immunostained with antibodies to ICAM1 (green) and Ki67 (white). Scale bar: 50 μm. (D) qRT‐PCR analysis of *Trp63* mRNA expression in the olfactory epithelial organoids untreated (control) or treated with H1N1, IL‐4, IL‐13, IFN‐γ, TNF‐α, IL‐1β and IL‐25. Data were collected from three independent experiments (*n* = 3) and are shown as means ± SD; One‐way ANOVA were performed. *p < 0.05, **p < 0.01, ***p < 0.001.

To explore the mechanisms underlying tuft cell hyperplasia observed in *Gng13*‐ and *Trpm5*‐deficient mice, particularly following H1N1 infection, we hypothesized that cytokines—elevated during inflammation—may activate otherwise quiescent HBCs to proliferate and differentiate. This hypothesis was supported by the increased immune infiltration and cytokine expression observed in the mutant olfactory tissues at both baseline and post‐infection (Figures 2 and 3). To further test this hypothesis, we employed an olfactory epithelial organoid system derived from ChAT‐Cre: Ai9 mice, which labels tuft cells with Ai9 (tdTomato), and treated the organoids with various cytokines (IL‐4, IL‐13, IL‐25, IFN‐γ, TNF‐α, IL‐1β) or H1N1 virus for 7 days (Figure 7A). Under control conditions, two distinct organoid morphologies were observed: large hollow spheres and smaller solid structures [54]. While most cytokine treatments did not alter the gross morphology of the organoids, IFN‐γ and TNF‐α had contrasting effects: the former induced the formation of predominantly small, solid organoids, whereas the latter favoured the development of large, hollow ones (Figure 7B). Notably, both IL‐4 and IL‐13 treatments led to a marked increase in tdTomato^+^ tuft cells, indicating enhanced stem cell differentiation.

To directly assess HBC proliferation, we immunostained organoid sections for ICAM1 and Ki67. Treatments with IL‐4, IL‐13 and IFN‐γ increased the number of ICAM1^+^Ki67^+^ cells compared with the untreated controls, reflecting robust activation of HBCs (Figure 7C). Complementary qRT‐PCR analysis revealed a significant downregulation of *Trp63* mRNA in these organoids (Figure 7D). Given that *Trp63* maintains HBC quiescence, its suppression is consistent with HBC activation and lineage progression [55, 56, 57]. Interestingly, cytokines exhibited distinct effects on *Trp63* expression: while IL‐1β and TNF‐α did not significantly alter *Trp63* levels, IL‐25 markedly upregulated *Trp63* expression, suggesting its possible role in preserving HBC dormancy.

Together, these results highlight the differential regulatory roles of cytokines in HBC fate determination. IL‐4 and IL‐13 not only stimulate proliferation but also promote HBC differentiation into olfactory tuft cells, paralleling mechanisms observed in intestinal tuft cell hyperplasia. IFN‐γ, while inducing proliferation, did not promote tuft cell differentiation and instead altered organoid morphology towards a regenerative, solid phenotype.

## 4 Discussion

Tuft cells have been found in various tissues [47]. Despite their diverse anatomical locations, these cells share a core gene expression profile, including genes encoding canonical gustatory signalling proteins such as Gα‐gustducin, Gγ13, Trpm5, as well as additional markers including Pou2f3, Lrmp, COX‐1 and IL‐25. Our data reveal that olfactory tuft cells also express many of these proteins; however, the commonly used marker Dclk1 is not detectable in olfactory tuft cells (Figures 1 and S1–S4) [4], a finding consistent with a previous study showing that DCLK1 is absent in human intestinal tuft cells as well [58]. This observation underscores the heterogeneity of tuft cells across different tissues and species. Moreover, tuft cells undergo complex maturation processes, which may further contribute to their diverse functions in inflammatory responses, inflammation resolution and subsequent functional recovery [58].

Gustatory signalling proteins in tuft cells have been implicated in the detection of and responses to pathogens [59]. In the present study, we generated Gng13‐cKO mice by crossing ChAT‐Cre mice with Gng13^loxP^ mice. Results from the ChAT‐Cre:Ai9 mice indicated that most of the ChAT‐expressing cells were tuft cells in the olfactory mucosa (Figures 1 and S1), whereas the scRNAseq data reanalysis showed that most *Gng13*‐expressing cells are tuft cells or olfactory sensory neurons (Figure S3). These results indicate that the conditional knockout of *Gng13* occurred selectively in olfactory tuft cells, although it cannot be excluded that a few other cells might also have been mutated.

Under homeostatic conditions, Gng13‐cKO and Trpm5‐KO mice showed increased numbers of CD45^+^ immune cells and GATA3^+^ ILC2 cells within the olfactory mucosa compared with WT controls (Figures 2 and 3). This finding suggests that olfactory tuft cells may play a role in maintaining the blood‐olfactory barrier, contributing to the regulation of immune homeostasis in the olfactory mucosa [17]. Further studies are needed to reveal whether disruption of the characteristic signalling proteins in these cells interrupts the communications between olfactory tuft cells and other mucosal cells such as ionocytes, sustentacular cells, olfactory sensory neurons and immune cells, or compromises the BOB function, thereby facilitating the invasion of inhaled microbes and other environmental insults into the olfactory tissue. Nevertheless, these mutations did not alter the numbers of F4/80^+^ macrophages, CD3^+^ T cells, Gsdmd^+^ or caspase‐1^+^ cells in the olfactory epithelium (Figures 2, 3, 4). Furthermore, the expression levels of the proinflammatory *Il6*, type 1‐associated *Tnfa* and *Ifng*, and type 2‐associated *Il13* and *Il25* cytokine genes remained unchanged (Figures 2 and 3). Therefore, the baseline inflammation associated with *Gng13* or *Trpm5* deficiency was limited and insufficient to trigger a broad immune response.

Olfactotropic pathogens are known to target multiple cell types within the olfactory epithelium [1]. Notably, viruses such as SARS‐CoV‐2 and influenza A have been shown to infect sustentacular cells and olfactory sensory neurons (OSNs), respectively, often resulting in significant pathological outcomes [10, 14, 15]. While OSNs have long been recognized as principal targets of influenza viruses [9], our findings show that H1N1 viruses can also infect olfactory tuft cells (Figure S5). Whether this infection results in tuft cell death or non‐lytic viral clearance remains to be elucidated. Nevertheless, this is, to our knowledge, the first report documenting direct infection of olfactory tuft cells by H1N1, offering new insights into H1N1 cell‐type tropism and possibly helping devise new strategies for preventing and mitigating H1N1 infection.

H1N1 infection evoked stronger immune responses from Gng13‐cKO and Trpm5‐KO than from WT olfactory mucosa. Disruption of *Gng13* and *Trpm5* resulted in modest baseline immune cell accumulation without triggering a full immune response. However, following H1N1 intranasal inoculation, both mutant mouse lines exhibited markedly exacerbated immune responses. An increased infiltration of CD45^+^ immune cells, including F4/80^+^ macrophages, GATA3^+^ ILC2s and CD3^+^ T cells, was found in the olfactory mucosa of Gng13‐cKO and Trpm5‐KO mice compared with those of WT control (Figures 2, 3, S6, and S7). Furthermore, increased apoptotic and pyroptotic cells were also observed in the mutant olfactory epithelia (Figures 4 and S8–S10). These results underscore the critical role of olfactory tuft cells in mediating pathogen‐induced immune responses and modulating the magnitude and kinetics of inflammatory responses.

In other tissues, tuft cells have been reported to function as frontline sentinels and detect invading parasites and microbial pathogens via G protein–coupled signalling pathways, triggering the release of diverse effector molecules, including acetylcholine (ACh), IL‐25, prostaglandins and leukotrienes. While some of these mediators amplify immune responses, others promote inflammation resolution. For example, intestinal tuft cells have been found to release acetylcholine, which acts on the muscarinic acetylcholine receptor on the adjacent epithelial cells, triggering epithelial chloride secretion, ultimately leading to helminth clearance [60, 61]. And indeed, single olfactory epithelial cell RNA‐seq data analysis indicated the expression of acetylcholine receptors and the chloride ion channel cystic fibrosis transmembrane conductance regulator (CFTR) in ionocytes [4]. Thus, it is possible that olfactory tuft cell‐derived acetylcholine may regulate the olfactory mucosa's immune response by altering ionocytes' function through acetylcholine. We therefore propose that loss of *Gng13* or *Trpm5* impairs the production of select tuft cell‐derived effector signals, thereby disrupting the balance between pro‐inflammatory and pro‐resolving cues. This imbalance may account for the disproportionate recruitment of particular immune cell subsets into the olfactory mucosa. Further studies are needed to validate this hypothesis.

Importantly, the pronounced increases in immune cell infiltration and cytokine expression in the mutant mice were most evident at 10–15 dpi—a period typically associated with tissue repair. These observations suggest that *Gng13* and *Trpm5* deficiencies may not only potentiate inflammatory responses but also hinder resolution mechanisms [39]. Identification of these mediators released by olfactory tuft cells may help design new ways to facilitate inflammation resolution and repair‐associated programmes.

H1N1 infection elevated the overall expression levels of cytokine genes *Il6*, *Tnfa*, *Ifng* and *Il4* in the olfactory mucosa of all genotypes with the exception of *Il4* in Gng13‐cKO, whereas *Il13* and *Il25* expression remained unchanged (Figures 2 and 3). However, distinct differences in cytokine expression were observed among these genotypes. Notably, *Il6* expression at 10 dpi was significantly higher in Gng13‐cKO mice compared with both WT and Trpm5‐KO mice, whereas *Ifng* expression was more pronounced in Trpm5‐KO mice. More strikingly, Gng13‐cKO completely abolished the H1N1‐induced increase in *Il4* expression, a response observed in WT and Trpm5‐KO mice. These findings suggest that Gγ13‐dependent pathways contribute to *Il4* induction in the infected olfactory mucosa, possibly by regulating tuft cell‐derived cues required for IL‐4 production by target cells. In contrast, the loss of *Trpm5*, a downstream effector ion channel in the Gγ13‐mediated signalling pathways, seems to have a more limited effect on tuft cell output signalling. Similar differences between Gng13‐cKO and Trpm5‐KO mice have been reported in the lung and gastrointestinal tract [39, 62], supporting the idea that tuft cells use multiple intracellular pathways to generate distinct output signals for neighbouring cells. Further investigations are warranted to elucidate the molecular mechanisms by which Gγ13‐mediated signalling regulates *Il4* expression.

HBCs are quiescent stem cells in the olfactory epithelium, and are typically activated after severe epithelial injury. Under homeostatic conditions, both Gng13‐cKO and Trpm5‐KO mice had more proliferating HBCs than WT mice. After H1N1 infection, proliferating HBCs increased significantly in WT mice, as expected, but the increase was more pronounced in Gng13‐cKO and Trpm5‐KO mice (Figure 6). One plausible explanation is that the disruption of the gustatory signalling pathways in olfactory tuft cells enhances immune cell infiltration into the olfactory mucosa, and these immune cells subsequently release signalling molecules that activate HBCs. Several mechanisms have been proposed for such activation, including olfactory tuft cell‐ACh‐ionocyte circuit [4] and the engagement of the NF‐κB/Stat6 pathway within HBCs, leading to the downregulation of the transcription factor p63, a repressor of HBC activation [63]. Other transcription factors including WNT, SOX2 and PAX6 may also contribute to this process [64]. Moreover, IL‐6, a cytokine significantly upregulated in the olfactory mucosa following H1N1 infection (Figure 2), has been shown to play a critical role in reactivating dormant cancer cells during viral infection [65], and may similarly promote HBC activation in the olfactory mucosa. Indeed, treatment of olfactory epithelial organoids with IL‐4, IL‐13 and IFN‐γ reduced *Trp63* expression and promoted HBC activation (Figure 7).

Activated HBCs can undergo symmetric and asymmetric cell division, leading to self‐renewal and differentiation into GBCs and other epithelial cells [66]. Our cell lineage tracing data indicated that following H1N1 infection, a subset of neurogranin‐expressing olfactory tuft cells were Ai14^+^, which appeared in the olfactory epithelium either individually or together with a group of other Ai14^+^ sustentacular cells, indicating that they were derived from Krt5^+^ HBCs, and that depending on the local microenvironments, either an individual or a few HBCs were activated (Figure S11). Immunostaining studies showed that some Pou2f3‐expressing tuft cells did not express the GBC marker Ascl1 (Figure S12), whereas some of the COX‐1^+^ tuft cells co‐expressed the HBC marker ICAM1 (Figure 6), suggesting that these tuft cells were not generated from GBCs, but instead, directly from HBCs. This result is consistent with a recent single‐cell transcriptomic study, which indicates that HBCs can directly differentiate into sustentacular cells [27]. Furthermore, our data show that the COX‐1^+^ ICAM1^+^ tuft cells were more abundant in Gng13‐cKO and Trpm5‐KO mice than in WT mice (Figure 6), implicating the possible regulatory role of olfactory tuft cells in HBC cell fate specification.

In the gastrointestinal tract, tuft cell–mediated type 2 immune responses are tightly coupled with stem cell activation and robust tuft cell hyperplasia [59]. However, unlike in the intestines [44], Gng13‐cKO and Trpm5‐KO mice showed increased olfactory tuft cell numbers even under homeostatic conditions (Figure 5). These differences may reflect the distinct environmental exposure of the olfactory epithelium, which is continuously exposed to inhaled pathogens and irritants; and Gng13‐cKO and Trpm5‐KO mice may therefore maintain a higher basal immune tone compared with WT mice. However, olfactory tuft cell expansion was limited in Gng13‐cKO and Trpm5‐KO mice after H1N1 infection, for which one possible explanation is that the baseline numbers of tuft cells in the mutant mice were too great to be further augmented following H1N1 infection. Together with the organoid data, our findings suggest that olfactory tuft cell expansion is regulated by IL‐4/IL‐13 signalling, supporting the presence of an IL‐25–ILC2–IL‐4/IL‐13 feedback loop in the olfactory epithelium. Moreover, tuft cell‐expressed IL‐17RB has recently been shown to be able to restrain IL‐25 bioavailability and limit tonic ILC2 stimulation [67]. A similar negative feedback mechanism may also exist in the olfactory mucosa to fine tune olfactory tuft cell expansion and mucosal immune responses. And loss of Trpm5‐ or Gγ13‐dependent signalling may compromise this regulatory circuit, allowing exaggerated ILC2 activation, cytokine production and immune cell recruitment.

## 5 Conclusions

Our data support a model in which olfactory tuft cells utilize Gγ13‐ and Trpm5‐dependent signalling pathways to regulate the release of mixed output signalling molecules including acetylcholine, IL‐25 and prostaglandins, which in turn act on other mucosal cells such as ionocytes, OSNs and immune cells to maintain the basal immune state of the epithelium as well as the quiescent state of HBCs. Disruption of these pathways alters the profile of the mixed output signalling molecules, leading to heightened infiltration of immune cells, which produce additional IL‐4, IL‐6, IL‐13 and other bioactive molecules that directly act on HBCs or alter the microenvironments for HBCs, resulting in augmented HBC activation and differentiation into sustentacular cells and tuft cells. Upon H1N1 infection, the imbalanced mixtures of the output signalling molecules from Gng13‐cKO and Trpm5‐KO olfactory tuft cells exacerbate immune responses, including increased immune cell recruitment, elevated cytokine production, enhanced cell death, altered inflammatory magnitude and accelerated replenishment of sustentacular cells, tuft cells and possibly other non‐neuronal epithelial cells (Figure S14). The rapid reconstitution of sustentacular cells and tuft cells may facilitate the reconstruction of the olfactory epithelium, but it occurs potentially at the expense of OSNs, as depletion of HBCs may compromise neurogenesis and ultimately impair olfactory functions. Together, our results reveal a previously unappreciated role of olfactory tuft cells in regulating and resolving inflammation, modulating HBC activation and differentiation. These findings provide new means for devising novel therapeutic interventions in olfactory disorders linked to chronic rhinosinusitis or post‐viral syndromes such as long COVID.

## Author Contributions

**Sai‐Sai Zhang:** conceptualization, data curation, formal analysis, validation, investigation, visualization, methodology, writing – original draft. **Haiyan Wang:** formal analysis, investigation, visualization, methodology, writing – original draft. **Yi‐Sen Yang:** formal analysis, investigation, visualization, methodology, writing – original draft. **Yi‐Hong Li:** investigation, methodology, writing – original draft. **Defu Yu:** investigation, methodology, writing – original draft. **Yushi Yao:** data curation, methodology, writing – original draft. **Ruhong Zhou:** conceptualization, supervision, funding acquisition, writing – review and editing. **Liquan Huang:** conceptualization, resources, data curation, formal analysis, supervision, funding acquisition, validation, visualization, methodology, writing – original draft, project administration, writing – review and editing.

## Funding

This work was in‐part supported by the Starry Night Science Fund of Zhejiang University Shanghai Institute for Advanced Study (SN‐ZJU‐SIAS‐005 to L.H., SN‐ZJU‐SIAS‐003 to R.Z.); National Key Research and Development Program of China (2021YFF1200803 to L.H., 2021YFF1200404 and 2021YFA1201200 to R.Z.); National Natural Science Foundation of China (U1967217 to R.Z.); National Center of Technology Innovation for Biopharmaceuticals (NCTIB2022HS02010 to R.Z.).

## Ethics Statement

All animal experiments were approved by the Institutional Animal Care and Use Committee (IACUC) of Zhejiang University (ZJU20240147) and performed following the NIH's ‘Guidelines for the Care and Use of Laboratory Animals’.

## Conflicts of Interest

The authors declare no conflicts of interest.

## Supporting information


**Figure S1:** Coexpression of molecular markers in olfactory tuft cells.
**Figure S2:** Olfactory epithelial organoids contain olfactory tuft cells.
**Figure S3:** Reanalysis of the scRNA‐seq dataset GSE245074 shows the expression of canonical tuft cell marker genes in type I olfactory microvillous cells.
**Figure S4:** Dclk1 is not a marker of olfactory tuft cells.
**Figure S5:** Influenza viruses infect tuft cells as well as other types of cells in mouse olfactory epithelium and olfactory epithelial organoids.
**Figure S6:** Both the disruption of the gustatory signalling pathway in olfactory tuft cells and H1N1 infection led to select immune cell accumulation in the olfactory tissue.
**Figure S7:** Both the disruption of the gustatory signalling pathway in olfactory tuft cells and H1N1 infection increase type 2 immune cells in the olfactory tissue.
**Figure S8:** The dysfunction of tuft cells aggravates cell death induced by influenza infection.
**Figure S9:** H1N1 infection increases cell death in the olfactory tissue of Gng13‐cKO and Trpm5‐KO mice.
**Figure S10:** H1N1 infection increases cell death in the olfactory tissue of Gng13‐cKO and Trpm5‐KO mice.
**Figure S11:** HBCs contribute to tuft cell generation following influenza virus infection.
**Figure S12:** A subset of tuft cells is derived directly from HBCs, instead of GBCs, following H1N1 infection.
**Figure S13:** Mature tuft cell marker neurogranin does not appear to be colocalized with the proliferating cell marker Ki67 before or after H1N1 infection.
**Figure S14:** A working model: possible roles of olfactory tuft cells in modulating baseline inflammation, coordinating immune response and HBC activation and differentiation following viral infection.
**Table S1:** Sequences of primers used for qPCR.

## Data Availability

All data generated or analyzed during this study are included in the article and Supporting Information. The following previously published dataset was used: GSE245074 from ‘S. Ualiyeva et al., “A Nasal Cell Atlas Reveals Heterogeneity of Tuft Cells and Their Role in Directing Olfactory Stem Cell Proliferation,” *Science Immunology* 9, no. 92 (2024): p.eabq4341’.
