# Supplementary material for: Olfactory Tuft Cells Are Critical to Basal Inflammation, Innate Immune Response to Viral Infection, and Modulation of Quiescent Stem Cell Activation, Proliferation and Differentiation
Source: Cell Prolif. 2026 Sep 19:e70281. Online ahead of print. doi: 10.1111/cpr.70281 (PMC13589258; doi:10.1111/cpr.70281)
Supplement: Supplementary file 1 [file CPR-9999-e70281-s001.docx]

Supplementary Material

# Supplementary Figures and Table

## 1. Supplementary Figures

**Supplementary Figure 1. Coexpression of molecular markers in olfactory tuft cells.**

(A) Among 565 Trpm5-lacZ^+^ cells, 499 or 88.3% were ChAT-Cre:Ai9^+^, whereas out of 516 ChAT-Cre:Ai9^+^ cells, 499 or 96.7% expressed Trpm5-lacZ.
(B) Among 462 Gα-gustducin (Gα-gust)^+^ cells, 444 or 96.1% were ChAT-Cre:Ai9^+^, whereas out of 506 ChAT-Cre:Ai9^+^ cells, 444 or 87.7% expressed Gα-gustducin.
(C) Among 472 COX-1^+^ cells, 471 or 99.9% were ChAT-Cre:Ai9^+^, whereas out of 496 ChAT-Cre:Ai9^+^ cells, 471 or 95% expressed COX-1.
(D) Among 544 Lrmp^+^ cells, 541 or 99.4% were ChAT-Cre:Ai9^+^, whereas out of 571 ChAT-Cre:Ai9^+^ cells, 541 or 94.7% expressed Lrmp.
(E) Among 534 neurogranin (Nrgn)^+^ cells, 528 or 98.9% were ChAT-Cre:Ai9^+^, whereas out of 550 ChAT-Cre:Ai9^+^ cells, 528 or 96% expressed neurogranin.
(F) Among 535 Pou2f3-CreERT2-IRES-EGFP^+^ cells, 514 or 96.1% were neurogranin (Nrgn)^+^, whereas out of 516 Nrgn^+^ cells, 514 or 99.6% expressed Pou2f3.

Supplementary Figure 2. Olfactory epithelial organoids contain olfactory tuft cells.

(A) Overlay images of red fluorescence and bright field of olfactory epithelial organoids obtained from ChAT-Cre:Ai9 mice and cultured for 10 days. Tuft cells expressing tdTomato (red) were found within the organoids. Scale bar: 100 µm.

(B-C) Representative images of the sections of olfactory epithelial organoids derived from ChAT-Cre: Ai9 mice stained with antibodies to Gα-gustducin (B) and IL-25 (C), respectively, both of which were co-localized with Ai9 (tdTomato, red). Scale bar: 50 µm.

**Supplementary Figure 3.** **Reanalysis of the scRNA-seq dataset GSE245074 shows the expression of canonical tuft cell marker genes in type I olfactory microvillous cells.**

(A) Clustering analysis of individual olfactory epithelial cells.

(B-L) *Gnat3* (B), *Gng13* (C), *Plcb2* (D), *Itpr2* (E), *Itpr3* (F), *Trpm5* (G), *Il25* (H), *Ptgs1* (I), *Il13ra1* (J), *Il4ra* (K) and *Il17rb* (L) were expressed in olfactory tuft cells.

**Supplementary Figure 4. Dclk1 is not a marker of olfactory tuft cells.**

(A) Representative images of the olfactory epithelium sections derived from the ChAT-Cre: Ai9 mice stained with antibodies to Dclk1. Dclk1 antibody largely stained the cells of Bowman’s glands, and did not overlap with Ai9. Scale bar: 50 µm.

**Supplementary Figure 5. Influenza viruses infect tuft cells as well as other types of cells in mouse olfactory epithelium and olfactory epithelial organoids**.

(A-B) Representative images of mouse olfactory epithelium sections infected with a high dose of 1,200 pfu (A) and a low dose of 120 pfu H1N1 virus (B), and stained with an antibody against the H1N1 influenza A viral nucleoprotein (H1N1-NP).

(C) H1N1-NP was co-localized with Ai9 (tdTomato, red) in the olfactory epithelium from the mouse infected with 120 pfu H1N1 viruses.

(D) Representative images of olfactory epithelial organoid sections infected with H1N1 viruses and stained with the antibody against H1N1-NP. The H1N1-NP immunostaining is colocalized with Ai9 to a tuft cell in an olfactory epithelial organoid. Scale bar: 50 µm.

**Supplementary Figure 6. Both the disruption of the gustatory signaling pathway in olfactory tuft cells and H1N1 infection led to select immune cell accumulation in the olfactory tissue**.

(A-D) Shown are the same images as those in Figure 2A, but with close-up insets. These are representative images of the olfactory epithelium tissue sections of WT, Gng13-cKO, Trpm5-KO mice at 0 dpi (A), 5 dpi (B), 10 dpi (C), and 15 dpi (D) post H1N1 infection, stained with antibodies against CD45 (red) and F4/80 (green), and DAPI for nuclei (blue). Scale bar: 50 µm.

**Supplementary Figure 7. Both the disruption of the gustatory signaling pathway in olfactory tuft cells and H1N1 infection increase type 2 immune cells in the olfactory tissue**.

(A-D) Shown are the same images as those in Figure 3A, but with close-up insets. These are representative images of the olfactory epithelium tissue sections of WT, Gng13-cKO, Trpm5-KO mice at 0 dpi (A), 5 dpi (B), 10 dpi (C), and 15 dpi (D) post H1N1-infection, stained with antibodies against GATA3 (red) and CD3 (green), DAPI for nuclei (blue). Scale bar: 50 µm.

**Supplementary Figure 8. The dysfunction of tuft cells aggravates cell death induced by influenza infection.**

(A) Representative images of the olfactory epithelium tissue sections of WT, Gng13-cKO, and Trpm5-KO mice at 0 and 10 dpi following H1N1 infection, stained with an antibody against cleaved caspase-3 (red), and DAPI for nuclei (blue). Scale bar: 50 µm.

(B) Statistical analysis of the total area of all cleaved caspase-3^+^ apoptotic cells over the total DAPI area. The area of apoptotic cells was unchanged in WT mice after influenza infection; however, it was significantly increased in Gng13-cKO and Trpm5-KO mice. Each dot represents a mouse; data are presented as means ± SD (n=4); Two-way ANOVA were performed. **P* <0.05, ***P* <0.01, ****P* <0.001.

**Supplementary Figure 9. H1N1 infection increases cell death in the olfactory tissue of Gng13-cKO and Trpm5-KO mice.**

(A-D) Shown are the same images as those in Figure 4A, but with close-up insets. These are immunostaining images of the olfactory epithelium tissue sections from WT, Gng13-cKO, and Trpm5-KO mice at 0 (A), 5 (B), 10 (C), and 15 dpi (D) following H1N1 infection with antibodies to the pyroptosis cell markers Gsdmd (red) and caspase-1 (green). Scale bar: 50 µm.

**Supplementary Figure 10. H1N1 infection increases cell death in the olfactory tissue of Gng13-cKO and Trpm5-KO mice.**

(A-B) qRT-PCR analysis of expression of the genes *Nlpr3* (A), and *Il1b* (B) of the pyroptosis pathway. Data are presented as means ± SD (n=3). Two-way ANOVA were performed. **P* <0.05, ***P* <0.01, ****P* <0.001.

**Supplementary Figure 11. HBCs contribute to tuft cell generation following influenza virus infection.**

(A) Experimental design for cell lineage tracing: Krt5-CreER:Ai14 mice received 5 doses of tamoxifen before H1N1 infection, and were sacrificed at 15 dpi for immunohistochemical analyses.

(B-D) Representative immunostaining images of the olfactory epithelial sections from the Krt5-CreER:Ai14 mice at 0 dpi (B), and at 15 dpi (C and D) showing the expression of neurogranin (Nrgn, green) and tdTomato (Ai14, red), with the nuclei stained with DAPI (blue). Note: neurogranin-expressing tuft cells appeared individually (C) or in a group with sustentacular cells. Arrows point to Ai14^+^neurogranin^+^ tuft cells. Scale bar, 50 µm.

**Supplementary Figure 12. A subset of tuft cells is derived directly from HBCs, instead of GBCs, following H1N1 infection.**

Representative immunostaining images of the olfactory tissue sections of Pou2f3-CreERT2-IRES-EGFP mice before (A, 0 dpi) and after (B, 7 dpi; C, 15 dpi) H1N1 infection. An Ascl1 antibody was used to label GBCs (red), whereas an EGFP antibody was used to amplify the EGFP fluorescent signal (green) of Pou2f3-expressing tuft cells. Both EGFP⁺Ascl1⁺ (arrows) and EGFP⁺Ascl1⁻ (arrowheads) cells were found at the bottom of the olfactory epithelia following H1N1 infection. Scale bar, 50 µm.

**Supplementary Figure 13. Mature tuft cell marker neurogranin does not appear to be colocalized with the proliferating cell marker Ki67 before or after H1N1 infection.**

(A) Double immunostaining with antibodies against neurogranin (red) and Ki67 (green) on the olfactory tissue sections from WT, Gng13-cKO and Trpm5-KO mice before or after H1N1 infection (10 dpi). No co-localization was observed. Scale bar: 50 µm.

**Supplementary Figure 14.** **A working model: possible roles of olfactory tuft cells in modulating baseline inflammation, coordinating immune response and HBC activation and differentiation following viral infection.**

(A-B) WT olfactory tuft cells sense inhaled pathogens and release a mixture of mediators to modulate the baseline inflammation of the olfactory mucosa. Disruption of the gustatory signaling proteins Gγ13 and Trpm5 partially disables olfactory tuft cells’ function, which may selectively affect some mediators’ release, causing a skewed immune response in the olfactory mucosa along with the biased recruitment of certain immune cells to the mucosa, leading to HBC proliferation and differentiation into tuft cells and other epithelial cells. H1N1 infection triggers a full-scale immune response from both WT and mutant olfactory mucosa. The mutant mice, however, seem to exhibit stronger immune responses, including more immune cell infiltration, heightened cell death, altered cytokine expression, enhanced HBC activation and differentiation.

## 2. Supplementary Table

| Supplementary Table 1. Sequences of primers used for qPCR | | |
| --- | --- | --- |
| Gene name | Sequences (5’-3’) | Sequences (5’-3’) |
| *Actb* | GAACCCTAAGGCCAACCGTGAAAAGAT | GCAGGATGGCGTGAGGGAGAGCA |
| *Il13* | TGAGCAACATCACACAAGACC | GGCCTTGCGGTTACAGAGG |
| *Tnfa* | AGCCCACGTCGTAGCAAACCAC | AGGTACAACCCATCGGCTGGCA |
| *Il25* | ACAGGGACTTGAATCGGGTC | TGGTAAAGTGGGACGGAGTTG |
| *Nlrp3* | CCCCTTTATTTGTACCCAAGGCT | GCAACGGACACTCGTCATCT |
| *Il1b* | CCTGCAGCTGGAGAGTGTGGAT | TGTGCTCTGCTTGTGAGGTGCT |
| *Ifng* | AGCTCTTCCTCATGGCTGTTTCT | GTTGTTGCTGATGGCCTGATTG |
| *Il6* | TCTGGGAAATCGTGGAAATGAGAAA | AGTTGTTCTTCATGTACTCCAGGT |
| *Il4* | CAACCCCCAGCTAGTTGTCATCC | ACAGACGAGCTCACTCTCTGTGG |
| *Trp63* | CCTCCAACACAGATTACCCG | CTTCGCAATCTGGCAGTACA |
